# Caecal perforation with faecal peritonitis – unusual presentation of Bochdalek hernia in an adult: a case report and review of literature

**DOI:** 10.1186/1749-7922-4-16

**Published:** 2009-05-06

**Authors:** Ameet Kumar, Vikas Maheshwari, TS Ramakrishnan, Samaresh Sahu

**Affiliations:** 1 Department of Surgery, Air Force Hospital,, Nathu Singh Road, Kanpur Cantt Uttar Pradesh, India; 2 Department of Radiology, Air Force Hospital,, Nathu Singh Road, Kanpur Cantt, Uttar Pradesh, India

## Abstract

The improper fusion of the postero-lateral foramen of the diaphragm was first described by Bochdalek in 1848. The incidence of congenital diaphragmatic hernia varies from1:2000 to 1:5000 live births and Bochdalek hernias (BH) account for 75 to 85% of these. Although it is a well-known entity in neonates, it is occasionally discovered incidentally in adult patients. Until now, a total of around 100 cases of occult asymptomatic Bochdalek hernia have been reported. The symptomatic cases are encountered more rarely. Colon necrosis among the symptomatic cases was reported in some reports. We discuss the present case since we believe it to be, to the best of our knowledge, the first case of a Bochdalek hernia in an adult presenting with caecal perforation and faecal peritonitis and review the published literature about this rare condition.

## Introduction

A diaphragmatic hernia may be congenital or secondary to a traumatic rupture of the diaphragm. The incidence of congenital diaphragmatic hernia (CDH) varies from1:2000 to 1:5000 live births [1]. Bochdalek hernias (BH) and Morgagni hernias (MH) account for 75 to 85% and 1 to 6% among causes of CDH, respectively. Most CDHs are diagnosed antenatally or in the neonatal period and only 5% of CDH present after neonatal period. Approximately, over 100 cases of occult Bochdalek hernias in asymptomatic adults have been reported in the literature [2,3]. According to a review report presented in 1995, there were only five previous cases in which the colon was found in the thorax [4]. A medline search has revealed only a few cases of colonic necrosis in symptomatic cases wherein primary colo-colonic anastomosis was employed [3]. Another case presenting with perforation of the transverse colon was managed with Video assisted thoracoscopic surgery (VATS) and laparotomy [5]. We herein report the present case since we believe it to be the first adult Bochdalek hernia presenting with perforation of the caecum and faecal peritonitis secondary to a closed loop obstruction and review the published literature.

## Case Report

**Figure 1:**
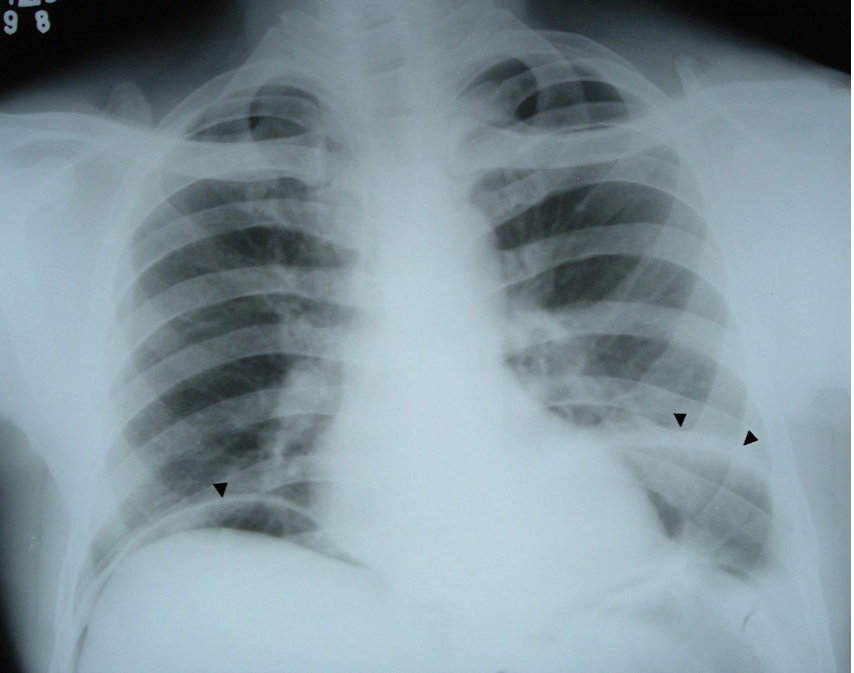
**Chest X-ray showing free air under diaphragm (single arrow head) along with the Bochdalek hernia on the right side (double arrow head)**.

**Figure 2:**
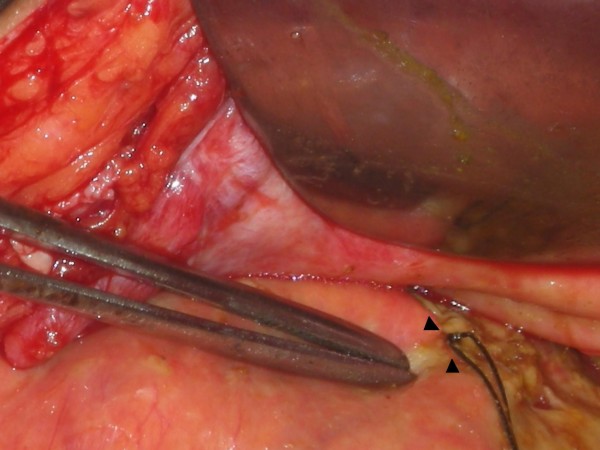
**Intraoperative picture showing markedly dilated caecum with perforation temporarily controlled by silk sutures**.

**Figure 3:**
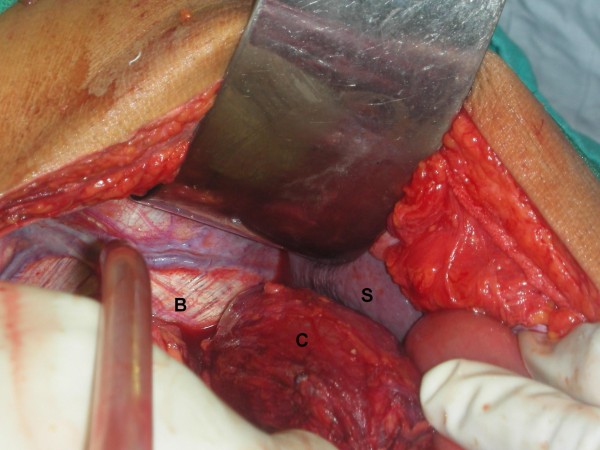
**Intraoperative picture showing transverse colon entering the posterolateral defect in the left diaphragm, B: Bochdalek hernia, S: Spleen, C: Transverse Colon**.

**Figure 4:**
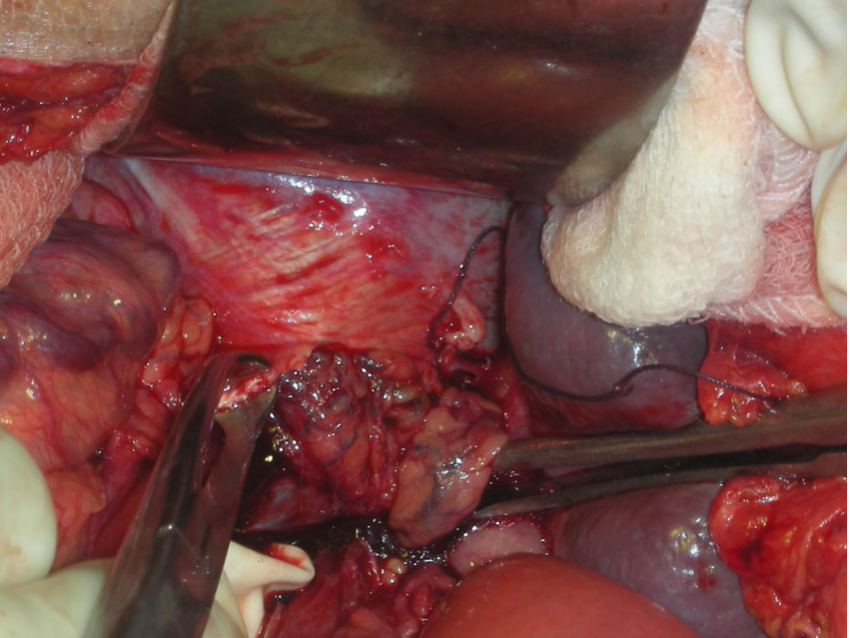
**Intraoperative picture of the defect having been enlarged to reduce the hernia**.

A 46-year-old male patient presented to our emergency department with a history of generalized abdominal pain of 7 days' duration. The pain had become more localized to the right lower abdomen for the last 2 days. There was a history of constipation lasting for 3 days. There was no vomiting and he did not have any chest or abdominal complaints in the past. There were no known co-morbidities. There was no history of recent trauma or surgery. On physical examination, he was febrile (101 Fahrenheit) and had tachycardia. Abdomen was distended and the liver dullness was obliterated. There was generalized abdominal tenderness in addition to rebound tenderness in the right iliac fossa. The bowel sounds were absent. The haemogram showed leucocytosis (11000/Cu mm). Chest X-ray showed free air under the diaphragm (Fig 1) and abdominal X-rays showed a markedly dilated transverse colon. A preoperative diagnosis of hollow viscus perforation with peritonitis was made and the patient was taken up for emergency laparotomy. On laparotomy, there was caecal perforation with faecal peritonitis (Fig 2). There was marked dilatation of the caecum, ascending colon and transverse colon up to the level of splenic flexure of the colon. The descending colon was collapsed and there was no mass or band causing the obstruction. The dilated transverse colon was followed and it became evident that it was entering the pleural cavity through a postero-lateral defect in the diaphragm (Fig 3). A dilated loop of transverse colon was found in the chest cavity with obstruction at the level of the defect. This loop along with its mesentery was viable and brought down into the abdominal cavity by enlarging the defect in diaphragm (Fig 4). The defect was primarily repaired in one layer with interrupted sutures of No-1 prolene and a left intercostal tube drain (ICD) with negative pressure was placed. The caecal perforation was managed by intracaecal placement of a Foley urethral catheter of 20 French to establish a tube caecostomy. In the postoperative period, ICD was removed on the 5th postoperative day. The patient developed mild infection at the laparotomy wound which was treated by conservative regimen. The caecostomy tube was removed after 3 weeks and the patient was subsequently discharged from the hospital.

## Discussion

Although the initial records of diaphragmatic hernia date back as far as the 1690s [6], the improper fusion of the postero-lateral foramina of the diaphragm was first described by Bochdalek in 1848 [7,8]. The true incidence of asymptomatic Bochdalek hernia remains unknown and ranges from 1/7,000 to 6% [7,9]. There is also reported predominance on the right side in asymptomatic cases [2]. Undiagnosed patients may never be identified as having Bochdalek hernia [2]. The left-sided presentation in our patient is in accord with the majority of cases reported in the literature. During the formation of the diaphragm, the pleural and coelomic cavities remain in continuity by means of the pleuroperitoneal canal. The posterolateral communication is the last to be closed by the developing diaphragm. Failure of the diaphragmatic development leaves a posterolateral defect symptomatic mostly on the left side. The defective closure of the pleuroperitoneal canal leads to three types of congenital hernias: the posterolateral (Bochdalek hernia), anterolateral and pars sternalis. The posterolateral subgroup accounts for 81% of these congenital diaphragmatic hernias [10]. Most of the Bochdalek hernias are diagnosed in children who present with pulmonary symptoms [6,7,11]. Since Bochdalek hernia in an adult is an asymptomatic condition, it is usually an incidental finding which makes its incidence difficult to be estimated. These can sometimes present with vague chest and gastrointestinal symptoms [6,11]. The predominance of the left side in symptomatic cases both in neonates and adults may be due to narrowing of the right pleuroperitoneal canal by the caudate lobe of the liver [12]. Another reason may be that the right pleuroperitoneal canal closes earlier. According to a recent report in 2002, there are only seven symptomatic cases involving the right hemidiaphragm in the literature [13]. The hernial size varies and the content of the hernial sac may differ from each other in every age group. Hernias on the left side may contain intestinal loops, spleen, liver, pancreas, kidney or fat. Colon in a Bochdalek hernia is a rare condition and usually found in the left-sided hernias as was also the case in our patient [7,14]. A medline search for cases of colon in a BH revealed about 32 cases (Table 1) [15-39]. A coexisting hernial sac has also been reported in 10–38% of the cases according to large series [7]. Some authors believe that long-term survival may be due to the persistence of a pleuroperitoneal sac (hernial sac) and that the rupture of the sac in adult life may trigger the characteristic symptoms [40]. There was no hernial sac in our patient. Drugs such as thalidomide or antiepileptics administered during pregnancy i.e. before the closure of the pleuroperitoneal canal before 9th to 10th weeks' gestation along with the genetic predisposition have been incriminated as the etiological factors. A congenital diaphragmatic hernia can be accompanied by other congenital anomalies in 25–57% and by chromosomal disorders in 10–20% of cases [10]. Our patient did not have any obvious congenital anomaly. Bochdalek hernias may show up on chest X-rays as air and fluid-filled viscera in the hemithorax, as in our case. Associated mechanical obstruction may be obvious on plain X-ray imaging. Contrast-enhanced computed tomography (CT) has been an increasingly important investigation method in assessment of acute presentation which was not used in our case. The rare finding of a dilated bowel above the hemidiaphragm makes the diagnosis obvious. Other investigations including upper gastrointestinal contrast studies can exclude malrotation [41]. Gastrointestinal contrast studies could not be done since our case was an emergency situation. A delayed or missed diagnosis of diaphragmatic hernia can lead to significant morbidity and mortality [42]. The diagnosis of Bochdalek hernia in adults is not easy, and on a couple of occasions has been misdiagnosed as pneumothorax and managed initially by a chest tube drainage resulting in feculent discharge from the chest and delayed discovery of hernia [5,43]. Management of a Bochdalek hernia includes reducing the abdominal contents and repairing the defect through a laparotomy or thoracotomy. The best approach for management of hernias occurring on the left side is controversial. Those who advocate a thoracotomy claim about the improved ability to separate adhesions between thoracic viscera and the hernial sac [42]. Those in favour of a laparotomy believe that the abdominal approach is superior to thoracotomy for the recognition and management of a possible concomitant malrotation and for dealing with visceral complications such as obstruction or strangulation [44]. Oliveira et al. favour a combined approach (laparotomy plus thoracotomy) for the right-sided cases to facilitate the replacement of the herniated viscera and to close the diaphragmatic defect to overcome the mass effect of the liver [45]. Our patient underwent an emergency laparotomy because of the presence of hollow viscus perforation with peritonitis. In the postoperative period, complications like abdominal compartment syndrome have been reported in literature following repair of an adult Bochdalek hernia [46,47]. The overall mortality in BH is around 12%. It is higher following emergency laparotomies (32%) than after elective surgery (3%) [48]. More recently, successful laparoscopic [49] and thoracoscopic repairs of the left sided Bochdalek hernia have both been described [5,50]. Some authors have also described hand assisted thoracoscopic repair of Bochdalek hernia [51]. Minimal invasive surgery is reported to be ideal for Morgagni defects, with a success rate of 90.9% with only one recurrence in a series, whereas it cannot be recommended in newborns with Bochdalek hernia because of high failure rates. It can be and should be considered for adults since the success rate increases with increasing age [52]. As our patient was operated on in a surgical emergency set-up caused by intestinal obstruction and hollow viscus perforation, a laparoscopic intervention was not possible.

**Table 1:** Summary of cases of Bochdalek hernia involving colon published in literature

Reference No	No of cases	Age	Sex	Presentation	Side	Operative Findings	Operative Procedure
15	1	76 y	M	Dyspnoea/intestinal obstruction	Right	Strangulation of a portion of transverse colon	Resection-anastomosis; primary repair
16	1	45 y	F	Pain abdomen	Right	Volvulus of colon	Right hemicolectomy; Primary repair
17	1	3 days	M	Respiratory distress	Right	Herniated small bowel, colon and liver	Thoracoscopic patch repair
18	1	Young	M	Abdominal pain	Left	Incarcerated colon	Primary repair
19	1	42 y	F	Abdominal pain, post prandial vomiting	Left	Sealed perforation of colon	Combined thoracoscopic and laparoscopic repair
20	1	16 y	M	Vomiting	Left	Stomach, spleen, part of the small intestine and colon in left hemithorax.	Primary repair
21	1	6 m	M	Bilious vomiting	Left	Herniated stomach, small intestine, part of colon and spleen	Laparoscopic repair
22	1	35 y	F	Intestinal obstruction	Right	Herniated colon with concurrent appendicitis	Appendicectomy, primary repair
23	1	2 y	M	Recurrent pulmonary infections	Right	Dilated colon loops and right kidney	Thoracotomy, primary repair
3	1	21 y	M	--	Left	Colonic necrosis	Resection anastomosis; primary repair
5	1	46 y	F	Abdominal pain, vomiting	Left	Colonic perforation (following chest tube insertion)	VATS combined with laparotomy; resection anastomosis with primary repair
24	1	25 y	F	--	Left	Herniated stomach, transverse colon, spleen	Laparoscopic repair
25	1	41 y	F	Abdominal pain, vomiting	Left	Herniated stomach, transverse colon, spleen	Primary repair
26	1	--	--	--	Right	--	--
13	1	63 y	F	Abdominal pain, dyspnoea	Right	Strangulated colon; kidney	Resection anastomosis; primary repair
27	1	Adult	F	Pain abdomen	Left	Gastric volvulus; colon	Laparoscopic repair
28	1	9 y	F	Cough, vomiting, anorexia	Right	Colon	Primary repair
29	2	Infants		Cough, vomiting	Right/Left	Colon, small intestine and stomach	Primary repair
30	1	49 y	F	Chronic cough	Left	Small intestine, colon	Hand assisted thoracoscopic prolene mesh repair
31	1	38 y	F	Abdominal pain, nausea	Left	Incarcerated transverse colon	Thoracotomy, primary repair
32	2	9 y	F	Abdominal pain, vomiting	Left	Small intestine, colon and stomach	Thoracotomy, primary repair
		35 y	M	Dyspnoea	Left	Small intestine, colon and stomach	Thoracotomy, primary repair
33	1	53 y	M	Intestinal obstruction	--	Obstructed colon	Primary repair
4	1	1 y	F	Fever, left pleural effusion, vomiting	Left	Colonic hernia	Primary repair
34	1	17 y	F	--	Left	Stomach, colon; hemorrhage	Primary repair
43	1	70 y	M	Dyspnoea	Left	Perforated colon following thoracic drain insertion	Combined thoracotomy and laparotomy; primary repair and colostomy
35	1	70 y	F	Abdominal pain, right chest pain	Right	Colopleural fistula due to Strangulated colon	Colostomy; primary repair
36	1	40 y	M	Mild nonspecific left chest pain	Left	Patient declined surgery	
37	1	5 y	M	Asymptomatic	Right	Stomach, colon	Primary repair
38	1	--	F	Abdominal pain, vomiting	--	Incarcerated colon	Primary repair
39	1	5 y	M	Dyspnoea, abdominal pain	Left	Herniated colon	Primary repair

Total number of cases: 32

## Conclusion

Bochdalek hernia is an uncommon variant of diaphragmatic hernias in adults and symptomatic cases are even rarer. Often, these cases present with chronic chest or abdominal symptoms and rarely present as an emergency like the present case with hollow viscus perforation and peritonitis. We want to emphasize the point that, though rare, diaphragmatic hernias should be kept in mind while considering all possibilities of the differential diagnosis of acute surgical abdomen in an adult, especially when a conventional plain X-ray reveals some abnormal findings.

## Competing interests

The authors declare that they have no competing interests.

## Authors' contributions

AK carried out the surgery, researched the article and drafted the manuscript.

VM assisted in the surgery, researched the article and drafted the manuscript.

TSR assisted in the surgery, edited and revised the manuscript.

SS carried interpreted the imaging studies, edited and revised the manuscript.

All authors read and approved the final manuscript.

## Consent

Written informed consent was obtained from the patient for publication of this case report and accompanying images. A copy of the written consent is available with the corresponding author for review by Editor-in Chief.
